# Transcriptome and MiRNAomics Analyses Identify Genes Associated with Cytoplasmic Male Sterility in Cotton (*Gossypium hirsutum* L.)

**DOI:** 10.3390/ijms22094684

**Published:** 2021-04-28

**Authors:** Min Li, Li Chen, Aziz Khan, Xiangjun Kong, Muhammad Rabnawaz Khan, Muhammad Junaid Rao, Jibin Wang, Lingqiang Wang, Ruiyang Zhou

**Affiliations:** 1 College of Agriculture, Guangxi University, Nanning 530004, China; lixiaominxy@sina.com (M.L.); junaidrao2012@gmail.com (M.J.R.); wangjibin1988@sina.cn (J.W.); 2 State Key Laboratory for Conservation and Utilization of Subtropical Agro-Bioresources, Guangxi University, 100 Daxue Rd., Nanning 530004, China; 3 Key Laboratory of Plant Genetic and Breeding, College of Agriculture, Guangxi University, Nanning 530005, China; jackliyi@sina.cn (L.C.); aziz.hzau@gmail.com (A.K.); rabnawazagri@gmail.com (M.R.K.); 4 School of Life Science and Technology, Henan Institute of Science and Technology, Xinxiang 453003, China; kongxiangjun201010@163.com

**Keywords:** upland cotton, pollen development, CMS, RNA sequencing, miRNA, ghi-MIR7484-10, *MAPKK6*

## Abstract

Cytoplasmic male sterility (CMS) is important for large-scale hybrid seed production. Rearrangements in the mitochondrial DNA (mtDNA) for the cotton (*Gossypium hirsutum L*.) CMS line J4A were responsible for pollen abortion. However, the expression patterns of nuclear genes associated with pollen abortion and the molecular basis of CMS for J4A are unknown, and were the objectives of this study by comparing J4A with the J4B maintainer line. Cytological evaluation of J4A anthers showed that microspore abortion occurs during meiosis preventing pollen development. Changes in enzyme activity of mitochondrial respiratory chain complex IV and mitochondrial respiratory chain complex V and the content of ribosomal protein and ATP during anther abortion were observed for J4A suggesting insufficient synthesis of ATP hindered pollen production. Additionally, levels of sucrose, starch, soluble sugar, and fructose were significantly altered in J4A during the meiosis stage, suggesting reduced sugar metabolism contributed to sterility. Transcriptome and miRNAomics analyses identified 4461 differentially expressed mRNAs (DEGs) and 26 differentially expressed microRNAs (DEMIs). Pathway enrichment analysis indicated that the DEMIs were associated with starch and sugar metabolism. Six deduced target gene regulatory pairs that may participate in CMS were identified, ghi-MIR7484-10/mitogen-activated protein kinase kinase 6 (*MAPKK6*), ghi-undef-156/agamous-like MADS-box protein AGL19 (*AGL19*), ghi-MIR171-1-22/SNF1-related protein kinase regulatory subunit gamma-1 and protein trichome birefringence-like 38, and ghi-MIR156-(8/36)/WRKY transcription factor 28 (*WRKY28*). Overall, a putative CMS mechanism involving mitochondrial dysfunction, the ghi-MIR7484-10/*MAPKK6* network, and reduced glucose metabolism was suggested, and ghi-MIR7484-10/*MAPKK6* may be related to abnormal microspore meiosis and induction of excessive sucrose accumulation in anthers.

## 1. Introduction

Cytoplasmic male sterility (CMS) is caused by genetic conflicts or communication barriers between nuclear genes and mitochondrial genes [1]. Mitochondrial genes can encode new proteins that interfere with mitochondrial function, leading to sterility and can also be silenced by nuclear restorer genes to reestablish fertility [2]. Mitochondrial DNA (mtDNA) is the carrier of CMS factors [3]. CMS genes in plants are mainly new open reading frames (ORFs) (chimeric regions) produced by frequent non-homologous recombination of mitochondrial genes with their flanking regions, chloroplast genes, or sequences of unknown origin [4,5]. Most of these chimeric genes encode ribosome [6,7,8,9,10] and mitochondrial electron transport chain complexes such as complexes IV [11,12,13,14,15] and V [12,13,16,17,18,19]. 

Loss of mtDNA function affects expression of nuclear genes by 4% [1,20,21]. Altered metabolic pathways influence the biological processes leading to CMS [7]. With the development of the technology for high-throughput sequencing and bioinformatics, CMS-related genes are discovered through the construction of transcription regulatory networks, which are related to the tricarboxylic acid (TCA) cycle, oxidative phosphorylation, respiratory electron transport chain, toxic proteins, and carbohydrate metabolism [22,23,24,25,26,27,28,29,30,31]. Besides, miRNA also plays an important role in flower development in plants [32]. Six miRNA-target gene pairs, miR160-*ARF*, miR156-*SPL*, miR159-*MYB*, miR164-*NAC*, miR172-*AP2,* and miR319-*TCP*, have been identified in maize sterile lines [33]. A regulatory network composed of miRNAs and target genes *ARF*, *MYB*, *bZIP*, *AP2,* and *TIR1*) participates in the development of flower buds, which finally leads to pollen abortion in citrus [34]. The results of miRNA and transcriptome analyses showed that the binding of hbr-mir156 and *SPL106* might be involved in the regulation of male sterility in cotton [35].

Cotton is an important economic crop and displays considerable heterosis. Utilization of cotton CMS germplasm has a significant impact on hybrid seed production. CMS lines of cotton were first developed in the 1960s, including CMS lines of *Gossypium arboreum* and B1-CMS lines of *Gossypium anomalum* [36,37]. Later, D2-2-CMS lines DES-HAMS16 and DES-HAMS277 of cotton were cultivated [38]. Since then, *Gossypium* *arboreum* A2-CMS line P24-6A, *Gossypium hirsutum* (AD)1-CMS line 104-7A, *Gossypium barbadense* (AD)2-CMS line Xiangyuan A, and *Gossypium trilobum* D8-CMS line D8ms have been bred successively [38,39,40,41]. However, cotton CMS germplasm resources are limited and have many negative effects, which has seriously restricted their utilization in the cotton industry.

CMS line J4A evaluated in this study was a new cotton CMS line bred by the transgenic method at Guangxi University [42,43]. The mtDNA for J4A was shown to be rearranged [9]. However, whether the rearrangement of mtDNA results in changes in mitochondrial activity and quantity and whether this consequently changes expression of any nuclear genes have not been determined. To further identify the mechanisms associated with sterility, this study compared the differences between the CMS line J4A and its maintainer line J4B to address the following three objectives: (1) the activity of mitochondrial respiratory chain complexes IV and V (MRCC IV and V) and the levels of ribosomal protein (RP) and adenosine triphosphate (ATP) in anthers before, during, and after pollen abortion was evaluated to determine their involvements in pollen development; (2) cytological observation was conducted and sugar metabolism was characterized to identify the pollen development stage associated with the onset of sterility, and (3) whole transcriptome sequencing for J4A and J4B at the meiosis period was performed and the negative regulatory relationship between mRNA and miRNA were evaluated to identify the mRNA–miRNA target gene regulatory pairs related to CMS. The results of this study will provide insights into the mechanisms underlying pollen abortion for J4A that will benefit the hybrid cotton industry.

## 2. Result

### 2.1. Morphological and Cytological Observations

**Figure 1:**
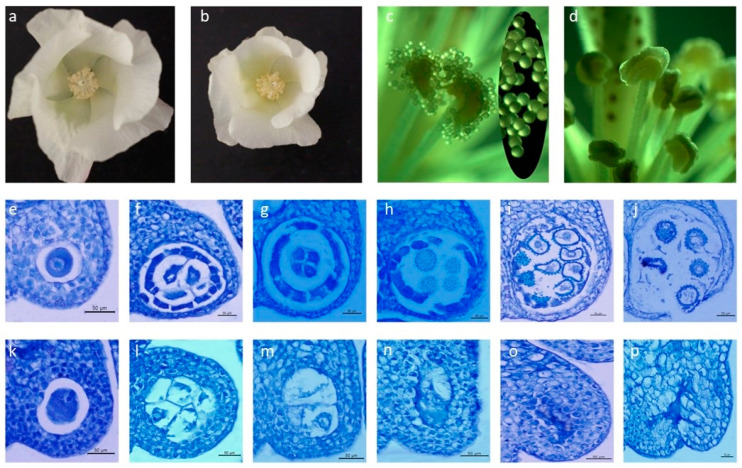
Morphology of CMS line J4A and maintainer line J4B: (**a**) flower of J4B, (**b**) flower of J4A, (**c**) anther of J4B with mature pollen grains shown in the oval insert, (**d**) anther of J4A, (**e**–**j**) anther of J4B, (**k**–**p**) anther of J4A, (**e**,**k**) pollen mother cell stage, (**f**,**l**) meiosis period, (**g,m**) tetrad period, (**h**–**j**,**n**–**p**) pollen ripening stage.

The corolla and ovary of J4A flowers were smaller and stamen filaments were shorter than those of J4B at anthesis (Figure 1a,b). Unlike J4B anthers, which had plump and cracked pollen grains (Figure 1c,d), J4A anthers were small, dry, and uncracked with no pollen grains observed. At the pollen mother cell stage, pollen sacs of J4A and J4B were surrounded by four cell structural layers (from the outside to the inside: epidermis, endothecium, middle epidermis layers, and tapetum cells), and no differences in development were observed between the lines at this stage (Figure 1e,k). At the start of meiosis, however, differences began to appear (Figure 1f,l). The middle cell layers for J4B pollen sacs began to degenerate, tapetum cells began to undergo mitosis, cytoplasm began to condense, and vacuolation occurred during meiosis, providing proteins, lipids, carbohydrates, and other nutrients for microspore development. These changes were not observed for J4A pollen mother cells. Tapetum cells developed slowly and middle cell layers showed no degradation. Additional developmental differences were observed during the tetrad stage; middle cell layers for J4B pollen sacs completely degenerated and pollen mother cells formed tetrads by meiosis that were surrounded by three cellular layers—the tapetum, endodermis, and exoderm. In comparison, tapetum cells for J4A showed no signs of degradation, pollen mother cells lacked the formation of tetrad structures, and pollen sacs began to shrink (Figure 1g,m). Tapetum cells of J4B continued to degrade, and pollen grains developed normally. However, pollen sacs for J4A became hard and irregular morphology was observed for the epidermis, inner layer, middle layer, tapetum, and pollen sac cavity with a lack of pollen grain development (Figure 1 h,n). At the pollen maturation stage for J4B, tapetal cells were completely degraded, pollen grains matured normally, and anther wall cracked for release of pollen grains. The disruption of pollen formation was clearly evident for J4A at this stage with distorted pollen sac structure, cells of each layer were irregularly arranged, and no pollen grains were present (Figure 1i,j,o,p). These results show expression of sterility occurring as early as the meiosis stage.

### 2.2. Mitochondria-Related Indexes

**Figure 2:**
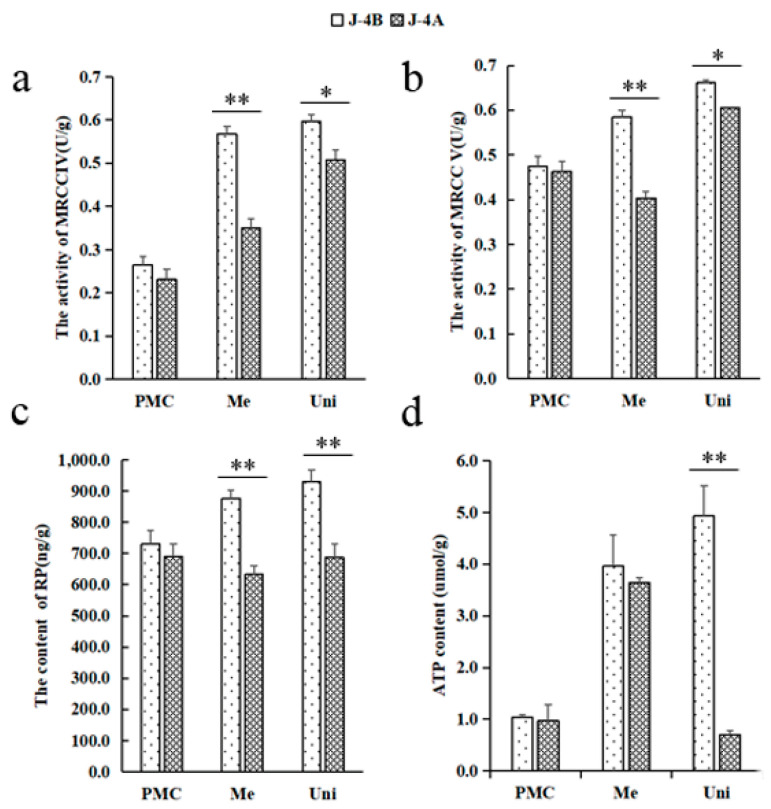
Mitochondrial biochemical indexes across different stages of anther development: (**a**) MRCC IV activity, (**b**) MRCC V activity, (**c**) RP content, (**d**) ATP content. *, significant difference (*t*-test, 0.01 < *p* < 0.05); **, high significant difference (*t* test, *p* < 0.01); PMC, pollen mother cell stage; Me, meiosis stage; Uni, uninucleate stage.

To detect whether the mitochondrial function was disrupted after mitochondrial genome rearrangement, four main mitochondrial function indexes were compared between J4A and J4B (Figure 2). Mitochondrial function indexes were similar at pollen mother cell stage for J4A and J4B, but changes were observed for J4A during the meiosis and mononuclear stages. Both J4A and J4B showed increases in MRCC IV activity at these later stages, but J4A showed a significantly lower increase in activity. MRCC V activity also increased during these stages for J4B; whereas, a decrease in activity was observed at the meiosis stage for J4A and the activity levels were significantly low at both stages compared to J4B. Lower RP content was observed during meiosis and mononuclear stages for J4A as compared to the pollen mother cell stage. For J4B, RP content showed a gradual increase across the three stages. ATP content showed a similar trend for J4B with an increase across the three stage. ATP content was highly variable across stages for J4A; however, a rapid decreased was observed at the monocyte stage (Figure 2d). These results indicate mitochondrial function was disrupted and could be detected during the meiosis stage. 

### 2.3. Overview of the mRNA and miRNA Sequencing Data for J4A and J4B

Anthers for J4A and J4B during meiosis (the abortion period) were collected, six mRNA libraries (accession number: PRJNA598065) and six miRNA libraries (accession number: PRJNA598474) were constructed (three replicates for each line), and an association analysis among mRNAs and miRNAs was performed. In total, 1,026,178,863 raw reads were obtained from the libraries. The RPKM (reads per kilobase per million mapped reads) was used to normalize the gene expression profiles to conduct comparisons among different genes and different groups. For the biological replicates, genes with *p* < 0.05 were considered differentially expressed genes (DEGs). Low-quality reads were filtered out, after which 981,063,720 clean reads were aligned to the *Gossypium hirsutum* L. reference genome (https://www.ncbi.nlm.nih.gov/assembly/GCF_000987745.1) (5 May 2015) (Supplementary Table S1). A total of 62,167 DEGs were detected. For the miRNA libraries, 149,851,709 raw reads were acquired. After the low-quality reads were filtered out, 113,637,852 miRNA reads were obtained. The sequence length distribution map shows that most of the clean reads of miRNAs were 18–24 nt in length; 24 nt was the most common length (Supplementary Figure S1).

### 2.4. Differentially Expressed (DE) miRNAs and mRNAs

**Figure 3:**
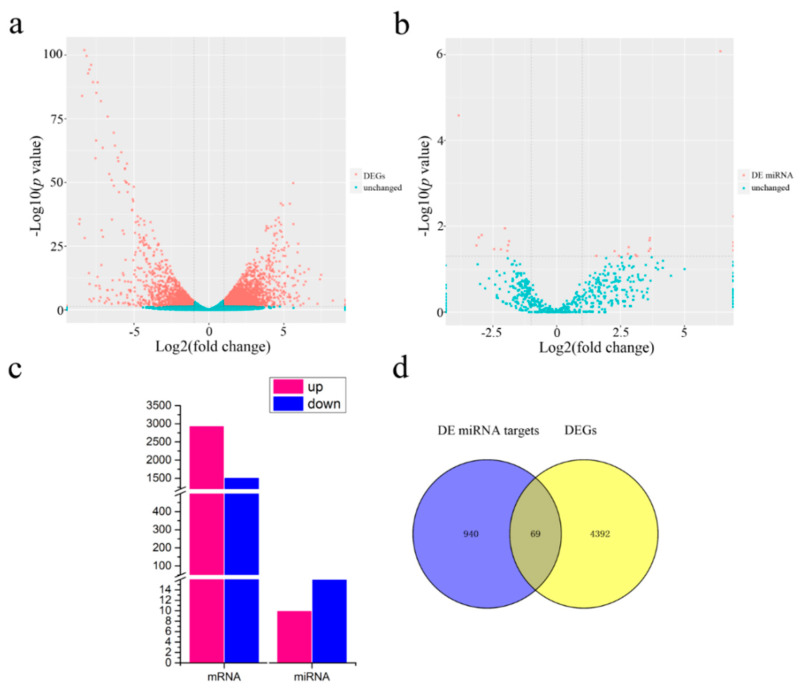
Statistics for the identified DEGs and DEMIs: (**a**) volcano maps of the DEGs between J4A and J4B, (**b**) volcano maps of the DEMIs between J4A and J4B, (**c**) statistics of DEGs and DEMIs, (**d**) Venn diagrams of the target genes of DEMIs and the DEGs.

RPKM was used to calculate the gene expression level. With screening criteria of a |fold change| > 2 and a significant *p*-value < 0.05, a total of 4461 DEGs was identified (Figure 3a,c), including 2940 upregulated and 1521 downregulated genes. The number of downregulated genes was significantly lower than that of upregulated genes. A total of 26 DE miRNAs (DEMIs) were identified using the same filtering criteria as for mRNAs (Figure 3b,c). Ten DEMIs were upregulated, and 16 were downregulated (Supplementary Table S2). There were significantly more downregulated than upregulated DEMIs. The DEMIs intersected with the DEGs to reflect changes in miRNA target genes, and 69 DE target genes of the DEMIs were identified (Figure 3d).

In the GO analysis of the molecular functions of DEGs (Supplementary Figure S2), most DEGs were associated with oxidoreductase activity (42%) (Supplementary Figure S3), showing that a change in redox homeostasis plays a significant role in the occurrence of CMS. Enrichment analysis of the DEGs revealed that the DEGs were involved in the plant hormone signal transduction, plant pathogen interaction, and glycolysis/gluconeogenesis pathways (Supplementary Figure S4). In the glycolysis pathway, the number of downregulated genes was remarkably higher than that of upregulated genes for J4A (Supplementary Figure S5). There were 256 genes involved in the glycolysis/gluconeogenesis pathway, among which 37 genes (14.3%) altered their expression models, including 21 downregulated genes and 16 upregulated genes. KEGG enrichment analysis of miRNAs showed that the DEMIs were most abundant in the starch and sucrose metabolism pathways (Supplementary Figure S6). Five DEGs were involved in sucrose synthesis; all of them were upregulated. Two DEGs participated in sucrose decomposition, and all of them were downregulated (Supplementary Figure S7). 

### 2.5. Regulatory Network of miRNAs and mRNAs

**Figure 4:**
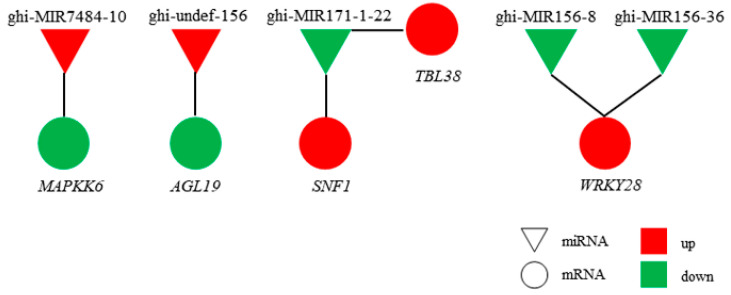
MiRNA–mRNA target gene regulatory networks: triangles represent miRNAs, circles represent mRNAs, red represents upregulated expression, and green represents downregulated expression.

In the network of genetic regulation in organisms, the role of transcription factors is to regulate mRNA transcription, while the function of miRNAs is to regulate the stability of mRNAs and translation after transcription. Based on the negative regulatory relationship between miRNAs and their target genes, 36 miRNA–mRNA target gene regulatory networks were obtained (Supplementary Table S3). Among the pairs, six are notable because they may be related to anther development (Figure 4). Specifically, the upregulated miRNAs—ghi-MIR7484-10 and ghi-undefined-156—inhibit the expression of *MAPKK6* and agamous-like MADS-box protein AGL19 (*AGL19*). Downregulated ghi-MIR171-1-22 promotes the expression of SNF1-related protein kinase regulatory subunit gamma-1 (*SNF1*) and protein trichome birefringence-like 38 (*TBL38*), and ghi-MIR156-8/ghi-MIR156-36 promotes the expression of WRKY transcription factor 28 (*WRKY28*).

### 2.6. QRT-PCR Validation of miRNA and mRNA Expression

**Figure 5:**
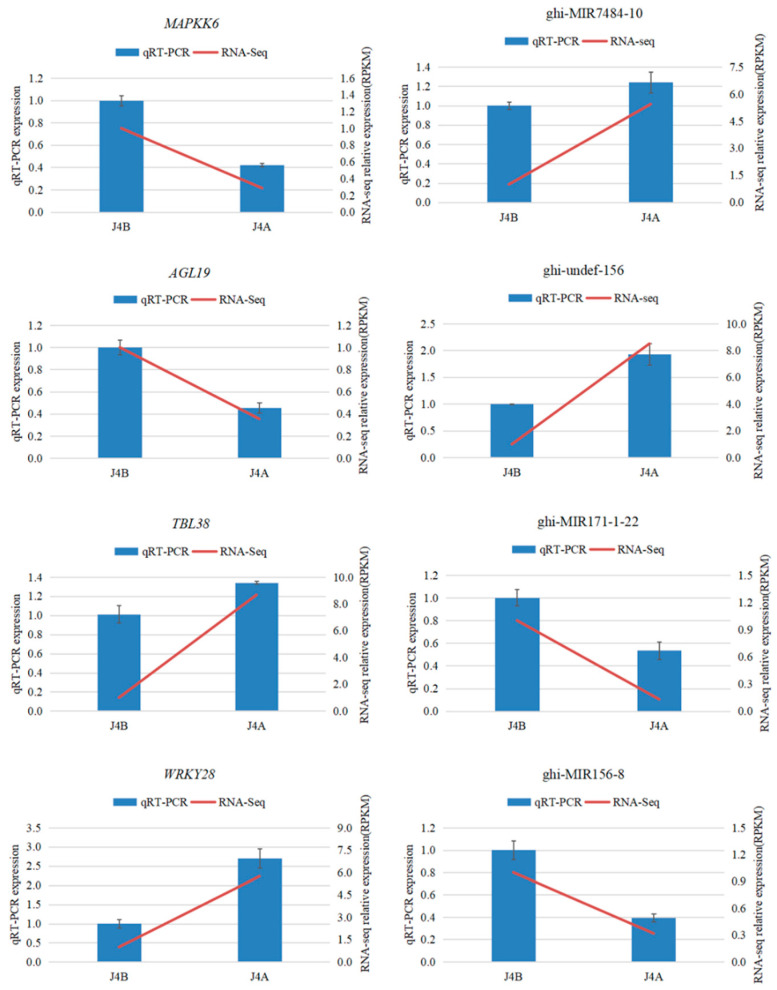
Validation of miRNA and mRNA expression patterns by qRT-PCR.

To verify the sequencing data, four miRNA–mRNA pairs were selected for expression pattern analysis by qRT-PCR (Figure 5). The relative expression levels of these selected miRNA–mRNAs were consistent with the trends in the RNA sequencing (RNA-Seq) data.

### 2.7. Determination of Physiological and Biochemical Indexes Related to the Glucose Metabolism Pathway

**Figure 6:**
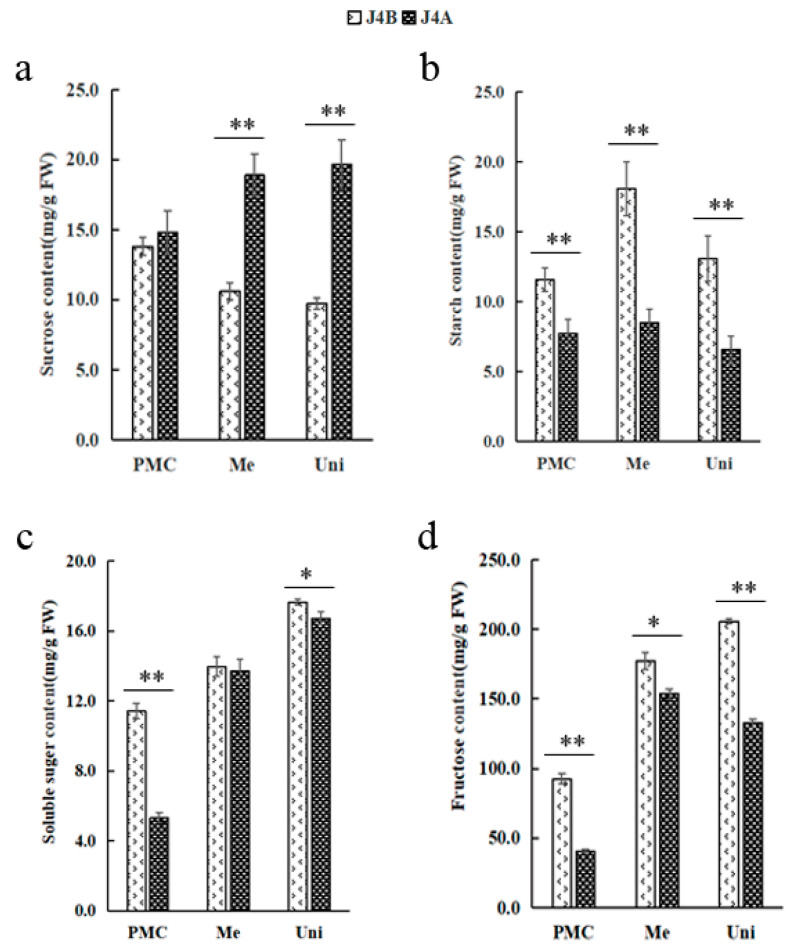
Physiological indexes of glucose metabolism in the CMS line J4A and the maintainer line J4B: (**a**) sucrose content, (**b**) starch content, (**c**) soluble sugar content, (**d**) fructose content. *, significant difference (*t* test, 0.01 < *p* < 0.05); **, very significant difference (*t* test, *p* < 0.01); PMC, pollen mother cell stage; Me, meiosis stage; Uni, uninucleate stage.

To determine whether the differential expression of genes involved in the glucose metabolism pathway had an effect, the levels of sucrose, starch, soluble sugar, and fructose were determined in anthers at three key stages of microspore development (Figure 6). Sucrose content increased across the developmental stages of J4A anthers; whereas, a decrease was observed across the three stages for J4B anthers with significant differences recorded between the lines at the meiosis and uninucleate stages (Figure 6a). In contrast, the levels of starch, soluble sugar, and fructose for J4A generally showed a similar pattern across the developmental stage as observed for J4B, but the levels were often significantly lower for J4A anthers. These results show a reduction in sucrose metabolism for J4A leading to higher levels during the meiosis stage and at later stages. 

## 3. Discussion

### 3.1. Period of Abortion in Cotton

Peak pollen abortion in dicotyledonous plants mostly occurs from the sporogenous cell stage to the tetrad stage [44]. Different species and even different CMS lines of the same cultivar may have different abortion modes and periods [45]. Pollen abortion for cotton CMS lines 12A, NM21A, and DE-HAMS227A develops primarily in the sporogenous cell proliferation or microsporocyte formation stages, and the main characteristics of abortion are mitosis of sporogenous cells and the formation of cells smaller than microsporocytes [46]. The main abortion stages of the Jin A cotton CMS line are the sporogenic cell and microspore mother cell, and the main characteristic is that the sporogenic cells cannot divide normally [44]. Pollen abortion for the cotton CMS lines H276A [22], LD6A [25], and C2P5A [27] occurs at the tetrad stage. Pollen abortion for the cotton CMS line J4A evaluated in the present study occurred during meiosis in the microspore mother cell stage, as the tapetum cells did not undergo programmed cell apoptosis during the entire developmental process, and the anthers ultimately had an insufficient energy supply and underwent abortion. The results are similar to the findings in a previous study which described the abortion characteristics for the Yamian A CMS line [45]. These reports showed that the mode of CMS expression in cotton is variable and characterization of these CMS systems would be useful for the development of hybrid cotton cultivars.

### 3.2. Disorder of Glucose Metabolism Is Related to the Occurrence of Male Sterility

Starch is the most important form of stored carbon, and its content in pollen gradually increases with another development and tends to be stable at the mature pollen stage; thus, starch content has often been used to determine pollen activity [46]. Obstruction of starch synthesis or excess decomposition reduces starch content in pollen, which eventually leads to sterility [47]. Sugar homeostasis is also essential [48,49,50] and abnormal glucose metabolism affects pollen and eventually leads to male sterility [48,51,52,53,54]. In Chinese cabbage, the levels of nonreducing sugars, reducing sugars, glucose, and fructose in male-sterile lines were shown to be significantly lower than in maintainer lines, which might have been due to differential expression of related genes [55]. Therefore, studying the physiological and biochemical metabolism would be important for revealing the mechanisms of CMS. The evaluation of CMS for J4A in the present study, shows that sucrose synthesis and hydrolysis were reduced, and starch content was decreased at meiosis, which led to an insufficient energy supply for another development. As a result, meiosis was inhibited (the tetrad structure could not be formed), which eventually led to pollen abortion. Consistent with these findings, the starch and sugar metabolic pathways were the most significant in the KEGG enrichment analysis in the present study. A total of 256 genes were involved in the glycolytic pathway, and 14.3% were DEGs. The proportion of downregulated genes among all DEGs was 2.8 times the proportion of upregulated genes among all DEGs. Five DEGs (LOC107958826, LOC107901030, LOC107893375, LOC107942366, and LOC107907080) were enriched in the sucrose synthesis pathway and upregulated; two DEGs (LOC107898531 and LOC107923558) were enriched in the sucrose decomposition pathway and downregulated. The differential expression of these genes is suggested for the decreased sucrose levels observed in anthers for J4A. It is concluded that sucrose accumulation is the main cause of glucose metabolism confusion. 

### 3.3. The MAPK Signaling Pathway Regulates the Process of Proliferation and Meiosis

Mutation of any core gene in the MAPK signaling pathway has been shown to lead to abnormal transition from the pachytene to diploid stage [56,57]. The generation of oocytes is driven by the MAPK signaling pathway in *Caenorhabditis elegans* gonads [56,58]. In the oocyte and germline-related 2 (*ogr-2*) mutant, the enhanced spatial activation of MAK-1 (MAPK terminal member) is related to the time of abnormal meiosis. It was inferred that through the spatial inactivation of MPK-1, OGR-2 can regulate the expression of lip-19 (a phosphatase that inhibits MPK-1), thereby promoting the timely progression of meiosis [59]. Pollen abortion observed for J4A in the present study was shown to result from abnormal meiosis of microspores. In addition, transcriptome and miRNAomics analyses identified ghi-MIR7484-10/*MAPKK6* as an important target pair related to CMS for J4A, which may contribute to the disruption of sucrose metabolism leading to the excessive accumulation of sucrose and abnormal microspore meiosis that results in pollen abortion.

## 4. Materials and Methods

### 4.1. Plant Materials

The cotton CMS line J4A (the mutant line) and maintainer line J4B (the wild type) were used [42,43]. Plants were grown in an experimental field at Guangxi University, Nanning, Guangxi Province, China, under natural conditions and normal field management. Sterile male plants were identified by observing the anther dehiscence and viable pollen grains, and their sterility was stable.

### 4.2. Morphological and Cytological Observations

Flower buds from J4A and J4B representing four major developmental stages (pollen mother cell, meiosis period, tetrad formation, and pollen ripening) were selected and classified according to the length of the buds. The classification unit was 0.5 mm. The buds were fixed in Carnoy fixative solution for 24 h, rinsed with 95% ethanol to remove glacial acetic acid, transferred to 70% ethanol, and stored at 4 °C [22]. The samples were next dehydrated through the following ethanol series: 50% ethanol→60% ethanol→70% ethanol→80% ethanol→85% ethanol→90% ethanol→95% ethanol→anhydrous ethanol. After vacuum drying for 30 min, the samples were kept at room temperature for 20 min. A clearing agent was used step-by-step and wax infiltration was carried out in an oven at 62 °C. The buds were then embedded in paraffin at 58–60 °C and sliced at a thickness of 8–10 µm. After dewaxing and hydration, the sections were stained with toluidine blue and then decolorized for observation under a microscope (DMI3000B, Leica, Wetzlar, Germany) and photographed. Flower morphology was conducted for flowers during anthesis and photographed.

### 4.3. Detection of Mitochondria and Glucose Metabolism Related Indexes

Anthers of J4A and J4B were collected (with three biological replicates) at the pollen mother cell stage (PMC; preabortion), meiosis stage (Me; abortion stage), and uninucleate stage (Uni; postabortion). A plant MRCC IV enzyme-linked immunosorbent assay (ELISA) kit, MRCC V ELISA kit, RP ELISA kit, and ATP kit (Shanghai Enzyme-Linked Biotechnology Co., Ltd., Shanghai, China) were used to measure mitochondrial function by the double antibody sandwich method [60]. The contents of starch, sucrose, soluble sugar, and fructose were determined (by visible spectrophotometry) with a plant sucrose content test kit (BC2465 Solarbio Technology Co., Ltd., Beijing, China), starch content test kit (BC0700, Solarbio Technology Co., Ltd., Beijing, China), soluble sugar content test kit (BC0030, Solarbio Technology Co., Ltd., Beijing, China), and fructose content test kit (BC2450 Solarbio Technology Co., Ltd., Beijing, China), respectively. The specific operation steps and calculation methods were conducted according to the kit instructions.

### 4.4. RNA Sequencing and Data Processing

Floral buds from J4A and J4B were collected during meiosis to represent the abortion period based on bud size (3.0–4.0 mm in length) and placed on ice. Anthers were peeled for buds on ice with RNA-free forceps and mixed as study materials. Two sets of three biological replicates were collected for each line with one set used for mRNA sequencing and the other set used for miRNA sequencing. The tissues were immediately frozen with liquid nitrogen and stored at −80 °C for further use. The ribosomal RNA (rRNA) was removed from total RNA with an rRNA removal kit, and RNA fragments of 200–300 nt were generated by ion interruption. RNA extraction was carried out by a Quick RNA Isolation Kit (Huayueyang Biotechnology Co., Ltd., Beijing, China). Library construction was accompanied by TruSeq^TM^ RNA Sample Preparation Kit v2 (Illumina, San Diego, CA, USA). Libraries were subjected to next-generation sequencing (NGS) in paired-end (PE) mode on an Illumina HiSeq 4000 sequencing platform. Low quality reads (quality score < 20) were filtered by trimmatic (version: v0.36). Using tophat2 (http://tophat.cbcb.umd.edu/) (25 April 2013), the filtered reads were compared to the *Gossypium hirsutum* reference genome (https://www.ncbi.nlm.nih.gov/assembly/GCF_000987745.1) (5 May 2015). Via HTSeq 0.6.1p2 (http://www-huber.embl.de/users/anders/HTSeq) (27 February 2014), the read count value of each gene was used as the original expression of the gene. RPKM (reads per kilo bases per million reads) was used to normalize gene expression. DESeq (version 1.18.0) (procmp, http://bioconductor.org/packages/release/bioc/html/DESeq2.html) (12 May 2014) was used to analyze the differences in gene expression. The thresholds for differential gene expression were as follows: a difference in expression (fold change) >2 and a *p*-value < 0.05. Volcano maps of differentially expressed genes (DEGs) were drawn by using the R language ggplot 2 software. TopGO (http://www.bioconductor.Org/packages/release/bioc/html/RamiGO.html) (15 July 2016) was used to map the gene ontology (GO) terms of the DEGs to a unique directed acyclic graph (DAG) structure, and the results were then organized according to the top GO terms. Small RNAs (miRNAs) were sequenced on a NextSeq 500 platform in single-end mode. Differences in miRNA expression were analyzed with DESeq (version 1.18.0) (procmp, http://bioconductor.org/packages/release/bioc/html/DESeq2.html) (12 May 2014). Conserved miRNAs and novel miRNAs were selected according to their fold changes in expression (|fold change| > 2) and the significance of their differential expression (*p* < 0.05). For sequences without any information, MIREAP was used to predict new miRNAs, and RNAfold was used to draw a secondary structure diagram.

### 4.5. Construction of miRNA–mRNA Regulatory Network

The target genes of miRNAs were identified by using psRNATarget [61] based on the known miRNAs, the newly predicted miRNAs and the gene sequence information of cotton. The predicted target gene sequences were compared with the GO database and the KEGG database with BLAST software, and the annotation information of the target genes was obtained. Based on the interaction between miRNA sequence and 3’UTR of mRNA and the principle of negative coexpression, the target mRNA genes of miRNA were predicted, and the interaction between mRNA and miRNA was found, that is, downregulation of miRNA/upregulation of mRNA and upregulation of miRNA/downregulation of mRNA. The intersection of differential miRNAs (target genes) and differential mRNAs was used for GO and KEGG functional enrichment analyses of candidate target gene set, and the regulatory network of differential miRNAs/differential mRNAs was constructed.

### 4.6. Verification of the Transcriptome and miRNA Sequencing Results by Quantitative Real-Time Fluorescence PCR (qRT-PCR)

Total RNA was extracted from anthers by the modified hexadecyltrimethylammonium bromide (CTAB) method. Single-stranded mRNA was transcribed into cDNA using a TransStart^®®^ Top Green qPCR SuperMix Kit (TransGen, Beijing, China). The 18S gene of cotton was used as the reference gene. Primers meeting the requirements of qRT-PCR (Supplementary Table S4) were designed online (http://unaford.rna.albany.edu/) (20 January 2006) and http://www.primer3plus.com/cgi-bin/dev/primer3plus.cgi) (26 January 2019). A miRNA qRT-PCR kit (Mir-X miRNA First-Strand Synthesis Kit (Clontech)) was used to verify the expression of miRNA according to the mature miRNA sequences, and ubiquitin 6 (U6) was used as the reference gene (Supplementary Table S4). All the reactions were performed with three replicates using CFX96 Real-Time PCR Detection System (Bio-Rad). The relative expression was calculated by the 2^−ΔΔCt^ method [62].

## 5. Conclusions

**Figure 7:**
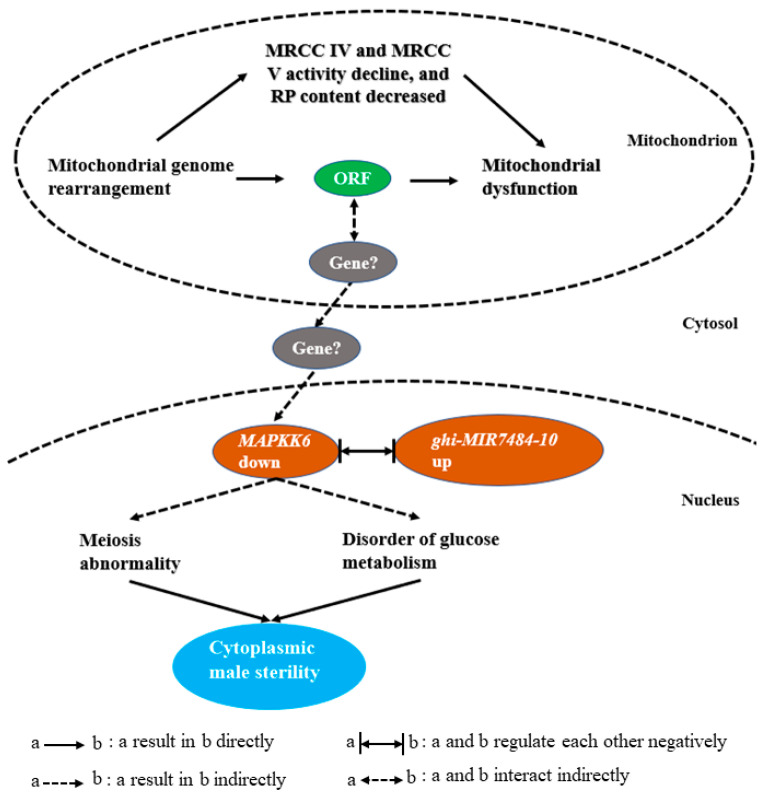
Schematic of a proposed mechanism for cotton CMS line J4A.

Mitochondrial dysfunction caused by mtDNA rearrangement in the cotton CMS line J4A results in pollen sterility. Data show that enzyme activity levels of MRCC IV and MRCC V were decreased, and RP content was also decreased, which led to insufficient synthesis of ATP in J4A anthers. Additionally, abnormal accumulation of sucrose and decreased starch content in J4A anthers were detected. Altered nuclear gene expression patterns (upregulation of ghi-MIR7484-10 resulted in downregulation of *MAPKK6*) were shown by miRNA–mRNA joint omics analysis. Microspore abortion was shown to occur during the meiosis stage preventing the formation of tetrad structures, which resulted in a lack of pollen production. The cotton anther miRNAs and RNAs identified in this study, combined with CRISPR/Cas9 technology, will be useful for the construction of male-sterile mutant libraries and to further explore the CMS mechanism for cotton. Although it is unclear how mitochondrial ORFs regulate the expression of nuclear genes and the occurrence of CMS, a hypothetical model is presented for the molecular mechanism of J4A CMS (Figure 7). 

## 6. Patents

Zhou Ruiyang, Li Min, A method of creating cytoplasmic male sterile line by transgenic cotton, 11 May 2020, China, ZL201711061141.8

## Supplementary Materials

The following are available online at https://www.mdpi.com/article/10.3390/ijms22094684/s1: Figure S1: The sequence length distribution of miRNA, Figure S2: GO analysis of molecular functions of DEGs, Figure S3: Molecular functions of the DEGs as revealed by GO analysis, Figure S4: KEGG enrichment analysis of the DEGs, Figure S5: Heatmap of the glycolysis pathway, Figure S6: KEGG pathway analysis of miRNAs in the CMS line J4A, Figure S7: Heatmap of the starch and sucrose pathway, Table S1: Statistics of the miRNA and mRNA sequencing data of J4A and J4B, Table S2: Detailed information on the top 16 upregulated and 10 downregulated miRNAs, Table S3: MiRNA–mRNA target gene regulatory pairs, Table S4: Primers of qRT-PCR.
