# Supplementary material for: Transcriptome and MiRNAomics Analyses Identify Genes Associated with Cytoplasmic Male Sterility in Cotton (Gossypium hirsutum L.)
Source: Int J Mol Sci. 2021 Apr 28;22(9):4684. doi: 10.3390/ijms22094684 (PMC8124215; doi:10.3390/ijms22094684)
Supplement: Supplementary file 1 [file ijms-22-04684-s001.zip › Supplementary Tables A.pdf]

**Supplementary Table S1**

Statistics of the miRNA and mRNA sequencing data of J4A and J4B

| | Type | J4A | J4B |
|---|---|---|---|
| | Raw reads | 434,942,372 | 441,384,782 |
| mRNAs | Clean reads | 430,439,840 | 436,986,028 |
| | Clean read % | 98.96 | 99 |
| | mRNAs | 62,167 | |
| | Raw reads | 69,327,639 | 80,524,070 |
| | Clean reads | 49,185,274 | 64,452,578 |
| miRNAs | Unique reads | 12,899,113 | 20,561,439 |
| | Conserved miRNAs | 62,956 | 274,335 |
| | Novel miRNAs | 9,875 | 14,082 |

**Supplementary Table S2**

Detailed information on the top 16 up-regulated and 10 down-regulated miRNAs

| ID | J4B RPKM | J4A RPKM | $Log_2$(fold change) | p-val | Regulation |
|---|---|---|---|---|---|
| ghi-undef-366 | 0 | 5.792377678 | - | 0.005890658 | up |
| ghi-undef-402 | 0 | 4.14178571 | - | 0.023840337 | up |
| ghi-MIR164-4 | 0 | 8.514523918 | - | 0.028682569 | up |
| ghi-undef-112 | 0 | 4.040286846 | - | 0.036246815 | up |
| ghi-undef-247 | 6.57816189 | 559.2783486 | 6.409738156 | 8.38E-07 | up |
| ghi-undef-286 | 5.42595457 | 69.06595539 | 3.670025866 | 0.033453193 | up |
| ghi-undef-329 | 5.739396465 | 72.19740999 | 3.652976142 | 0.018802719 | up |
| ghi-MIR8674-3 | 22.10800128 | 274.3479711 | 3.633366401 | 0.021313734 | up |
| ghi-undef-274 | 0.397173405 | 4.845444977 | 3.608788234 | 0.037878159 | up |
| ghi-MIR7494-1 | 18.38174832 | 162.5480187 | 3.144520076 | 0.049689517 | up |

| | | | | | |
|---|---|---|---|---|---|
| ghi-undef-156 | 10.41542284 | 88.61197648 | 3.088780291 | 0.046509228 | up |
| ghi-MIR7486-7 | 5.753338565 | 40.62567599 | 2.819920543 | 0.030575922 | up |
| ghi-MIR8674-1 | 5.997600586 | 41.28574665 | 2.783186443 | 0.043863191 | up |
| ghi-MIR7484-10 | 10.60513371 | 57.64723496 | 2.4424886 | 0.048856366 | up |
| ghi-undef-259 | 12.12464746 | 58.40728532 | 2.268205533 | 0.038042593 | up |
| ghi-MIR390-1 | 234.1766906 | 686.7774391 | 1.552245167 | 0.048809194 | up |
| ghi-undef-222 | 335.1554302 | 23.51607675 | -3.833111012 | 2.61E-05 | down |
| ghi-undef-325 | 15.87436733 | 1.798336247 | -3.141964393 | 0.02789001 | down |
| ghi-MIR171_1-21 | 23.2157918 | 2.803835297 | -3.049632976 | 0.018237255 | down |
| ghi-MIR171_1-22 | 34.60582459 | 4.53128426 | -2.933022979 | 0.015997398 | down |
| ghi-MIR171_1-10 | 43.15720756 | 7.90932519 | -2.447975003 | 0.034086607 | down |
| ghi-MIR156-36 | 133.3452977 | 29.41250316 | -2.180665475 | 0.034494997 | down |
| ghi-MIR156-8 | 1167.816527 | 287.1873341 | -2.023749605 | 0.0111208 | down |
| ghi-undef-215 | 380.9918972 | 100.0102848 | -1.929611944 | 0.037517776 | down |
| ghi-undef-495 | 847.4236732 | 226.9985585 | -1.900400295 | 0.027456708 | down |
| ghi-MIR166-15 | 4129.406964 | 1134.188268 | -1.864274469 | 0.022129182 | down |

**Supplementary Table S3**

MiRNA-mRNA target gene regulatory pairs.

| miRNA | mRNA | Gene | Annotation | miRNA regulation | mRNA regulation |
|---|---|---|---|---|---|
| ghi-MIR164-4 | gene2947 | LOC107892701 | ankyrin repeat domain-containing protein 13B-like | S_up | F_up |
| ghi-MIR164-4 | gene80 | LOC107897866 | uncharacterized LOC107897866 | S_up | F_up |

| | | | | | |
|---|---|---|---|---|---|
| ghi-MIR164-4 | gene83 | LOC107898061 | uncharacterized LOC107898061 | S_up | F_up |
| ghi-MIR7484-10 | gene66543 | LOC107932209 | mitogen-activated protein kinase kinase 6-like MAPKK | S_up | F_up |
| ghi-MIR7494-1 | gene34590 | LOC107897201 | mitochondrial arginine transporter BAC2-like | S_up | F_up |
| ghi-MIR8674-3 | gene17450 | LOC107956798 | uncharacterized LOC107956798 | S_up | F_up |
| ghi-undef-156 | gene9678 | LOC107946987 | agamous-like MADS-box protein AGL19 | S_up | F_up |
| ghi-undef-247 | gene57922 | LOC107924800 | E3 ubiquitin protein ligase DRIP2-like | S_up | F_up |
| ghi-undef-366 | gene20742 | LOC107960817 | uncharacterized LOC107960817 | S_up | F_up |
| ghi-MIR156-36/8 | gene52076 | LOC107916259 | uncharacterized protein At5g05190 | F_up | S_up |
| ghi-MIR156-36/8 | gene69845 | LOC107935885 | probable WRKY transcription factor 28 | F_up | S_up |
| ghi-MIR156-8 | gene43509 | LOC107906859 | homeobox protein knotted-1-like 3 | F_up | S_up |
| ghi-MIR166-15 | gene33424 | LOC107895419 | protein ABSCISIC ACID-INSENSITIVE 5-like | F_up | S_up |
| ghi-MIR166-15 | gene44631 | LOC107907684 | protein ABSCISIC ACID-INSENSITIVE 5-like | F_up | S_up |
| ghi-MIR166-15 | gene62048 | LOC107927187 | respiratory burst oxidase homolog protein B-like | F_up | S_up |
| ghi-MIR166-15 | gene62870 | LOC107928148 | protein ABSCISIC ACID-INSENSITIVE 5-like | F_up | S_up |

| | | | | | |
|---|---|---|---|---|---|
| ghi-MIR171_1-10 | gene27318 | LOC107888661 | probable galactinol--sucrose galactosyltransferase 5 | F_up | S_up |
| ghi-MIR171_1-10/21 | gene5589 | LOC107924044 | scarecrow-like protein 6 | F_up | S_up |
| ghi-MIR171_1-10/21 | gene5846 | LOC107926170 | scarecrow-like protein 6 | F_up | S_up |
| ghi-MIR171_1-10/21 | gene7945 | LOC107945536 | scarecrow-like protein 6 | F_up | S_up |
| ghi-MIR171_1-22 | gene12639 | LOC107950469 | probable trehalose-phosphate phosphatase J | F_up | S_up |
| ghi-MIR171_1-22 | gene47837 | LOC107912056 | probable LRR receptor-like serine/threonine-protein kinase | F_up | S_up |
| ghi-MIR171_1-22 | gene49468 | LOC107913418 | SNF1-related protein kinase regulatory subunit gamma-1 | F_up | S_up |
| ghi-MIR171_1-22 | gene58623 | LOC107923473 | probable polygalacturonase | F_up | S_up |
| ghi-MIR171_1-22 | gene59914 | LOC107925238 | protein trichome birefringence-like 38 | F_up | S_up |
| ghi-undef-215 | gene24718 | LOC107963891 | uncharacterized LOC107963891 | F_up | S_up |
| ghi-undef-215 | gene28616 | LOC107889687 | trafficking protein particle complex subunit 4-like | F_up | S_up |
| ghi-undef-215 | gene58678 | LOC107924387 | transmembrane protein 120 homolog | F_up | S_up |
| ghi-undef-222 | gene10734 | LOC107948534 | uncharacterized LOC107948534 | F_up | S_up |
| ghi-undef-222 | gene10902 | LOC107948638 | protochlorophyllide reductase, chloroplastic-like | F_up | S_up |

| | | | | | |
|---|---|---|---|---|---|
| ghi-undef-325 | gene49893 | LOC107913805 | probable inositol transporter 2 | F_up | S_up |

**Supplementary Table S4**

Primers of qRT-PCR

| Gene | Forward primer sequences | Reverse primer sequences |
|---|---|---|
| U6 | CATTTCTCGATTGTGCGTC | GGGGACATCCGATAAAATTGG |
| 18S | ATCAGCTCGCGTTGACTACGT | ACACTTCACCGGACCATTCAAT |
| ghi-MIR7484-10 | CCGCTTTGATCTAGTGTATGGAGACT | |
| MAPKK6 | AGAAACCGCCGAAGCCACTGA | TGAAAGGCGCAGGATCCCGA |
| ghi-undef-156 | CCTGGGATCTTCGAGAGAATTGA | |
| AGL19 | GCGTGAAGAGTGTGGGATGCGA | TCTTCTCTCCGGTGGGCCAA |
| ghi-MIR171_1-22 | CCAGATATTGGTGCGGTTCAATT | |
| TBL38 | GGCAAAATGGAGGGGAAAGAAA | GAGTTGGGCACAGAAGCATGAA |
| ghi-MIR156-8 | GGGCTCTCTATGCTTCTGTCATC | |
| WRKY28 | AGCAGCACTGTTCCCGCCTT | GCGCCGCCATCTGCATAAACA |
