# Supplementary figures and images for: Transcriptome and MiRNAomics Analyses Identify Genes Associated with Cytoplasmic Male Sterility in Cotton (Gossypium hirsutum L.)
Source: Int J Mol Sci. 2021 Apr 28;22(9):4684. doi: 10.3390/ijms22094684 (PMC8124215; doi:10.3390/ijms22094684)

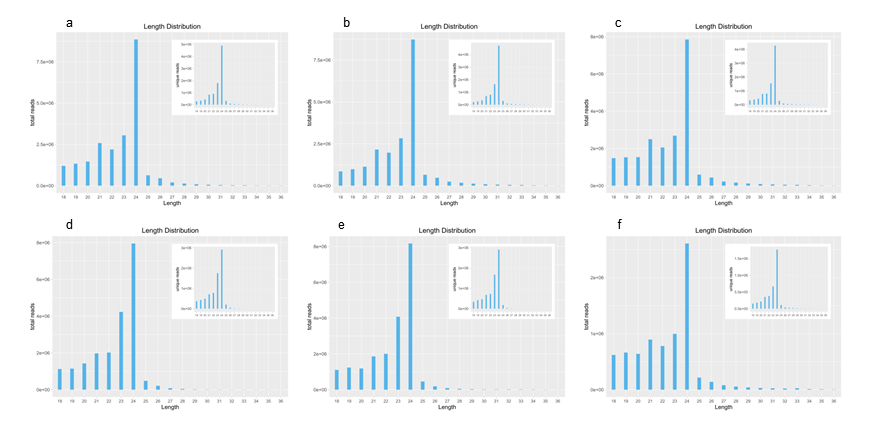

Supplement: Supplementary file 1 [file ijms-22-04684-s001.zip › Supplementary Figure S1.tif]

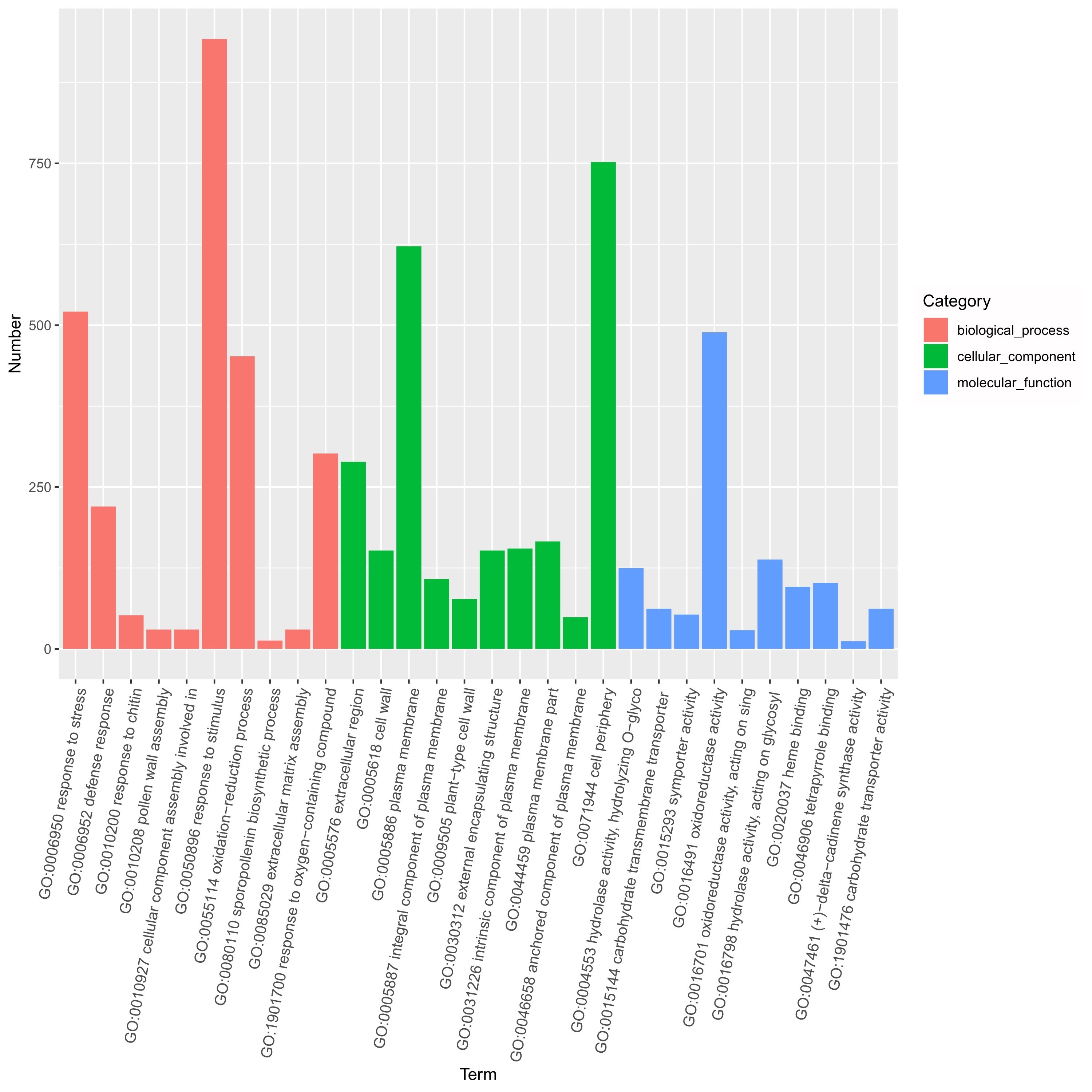

Supplement: Supplementary file 1 [file ijms-22-04684-s001.zip › Supplementary Figure S2.jpg]

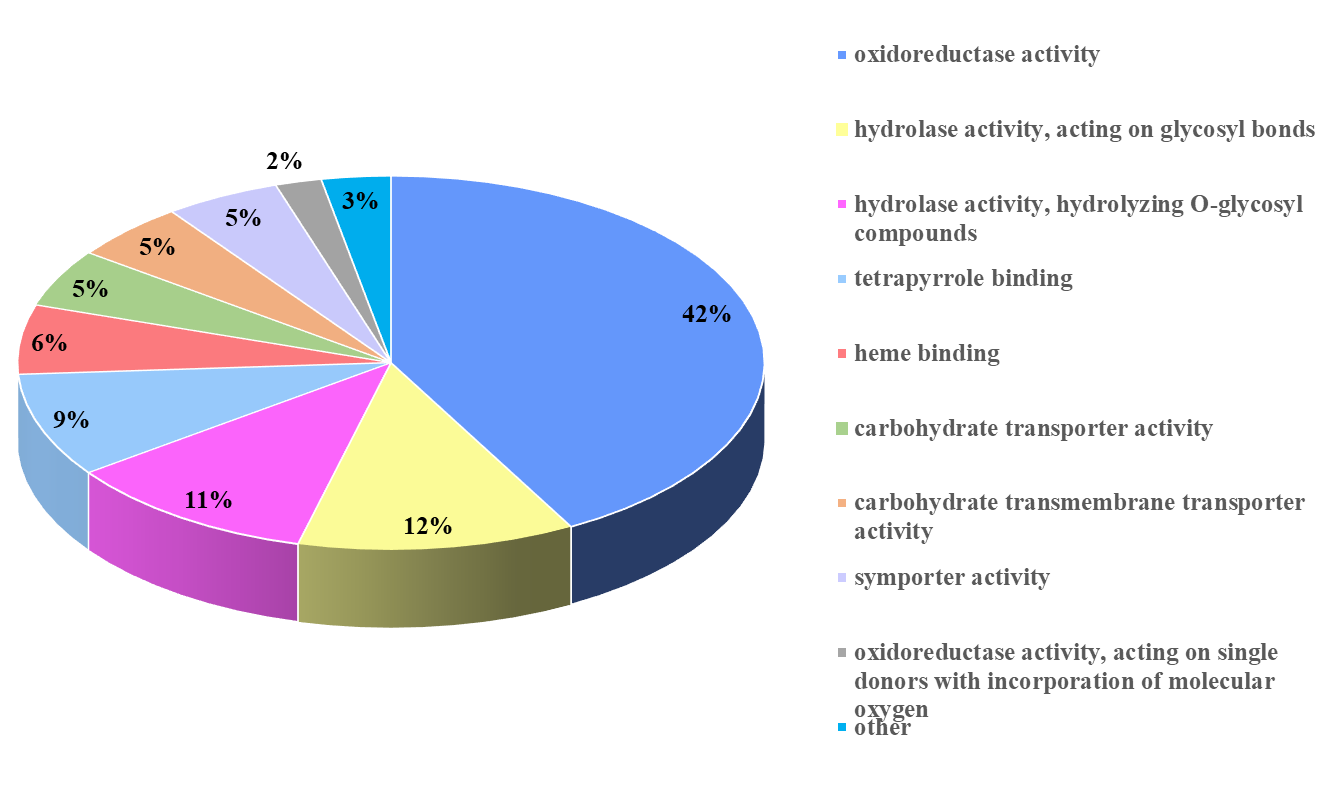

Supplement: Supplementary file 1 [file ijms-22-04684-s001.zip › Supplementary Figure S3.png]

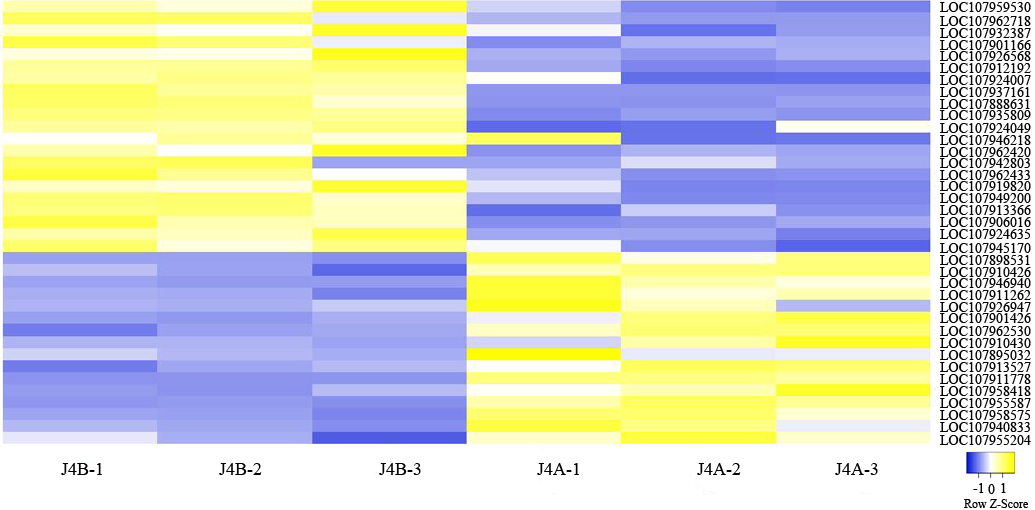

Supplement: Supplementary file 1 [file ijms-22-04684-s001.zip › Supplementary Figure S5.tiff]

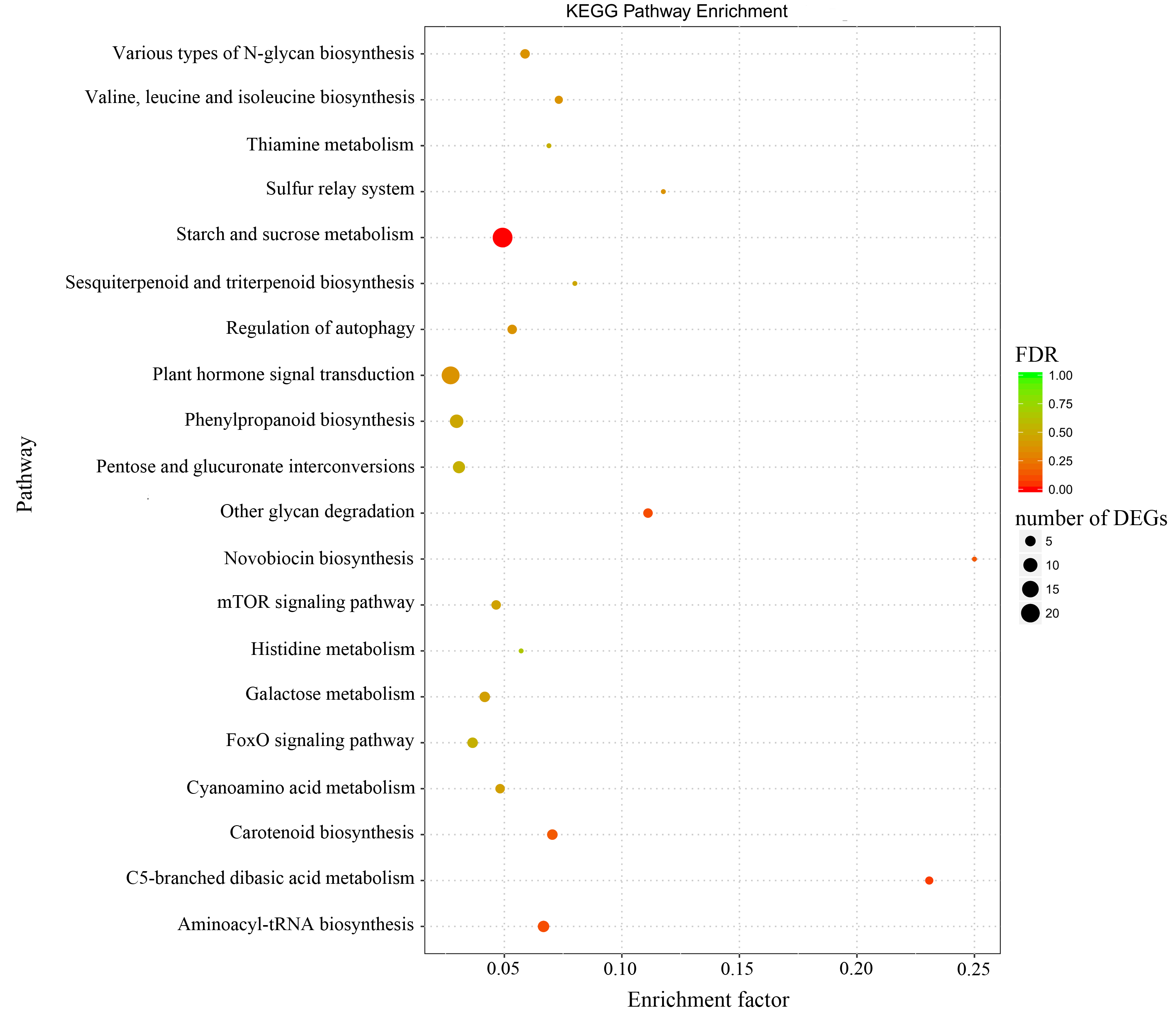

Supplement: Supplementary file 1 [file ijms-22-04684-s001.zip › Supplementary Figure S6.tif]

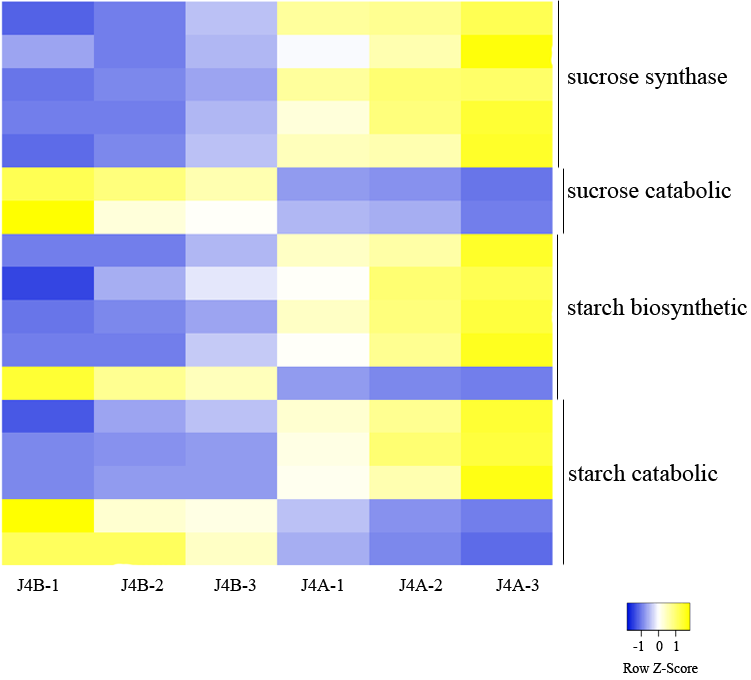

Supplement: Supplementary file 1 [file ijms-22-04684-s001.zip › Supplementary Figure S7.tif]
